# Expelled uninsured patients in a less-competitive hospital market in Florida, USA

**DOI:** 10.1186/s12939-016-0375-z

**Published:** 2016-06-04

**Authors:** Keon-Hyung Lee, Seunghoo Lim, Jungwon Park

**Affiliations:** Askew School of Public Administration and Policy, Florida State University, Tallahassee, FL 32306 USA; Public Management and Policy Analysis Program, International University of Japan, Minami Uonuma-shi, Niigata 949-7277 Japan; Department of Regulatory Research, Korea Institute of Public Administration, Eunpyeong-gu, Seoul 03367 South Korea

**Keywords:** Uninsured, Patient transfers, Hospital competition

## Abstract

**Background:**

This research evaluates the effect of hospital competition on inward and outward patient transfers for different types of payers including the uninsured. Although it is a less spotlighted issue, an equally important topic is the likelihood of inter-hospital patient transfers of the insured and the uninsured. This study attempts to fill a gap in the research about the relationship between hospital competition and patient transfers.

**Methods:**

By developing the payer-specific level of hospital competition, this research evaluates the effect of hospital competition on inward and outward patient sharing (or patient transfers) for different types of payers including the uninsured. For patient transfers, instead of focusing on whether a patient is transferred from one hospital to another hospital at the patient level, we measure the numbers of patient transfers between hospitals (both inward and outward) at the hospital level. These dependent variables—the numbers of outward and inward patient transfers by the principal payers—are count variables, and we employ either a Poisson regression model or a negative binomial regression model.

**Results:**

Controlling for hospital characteristics, when the uninsured Hirschman-Herfindahl Index (HHI) increased by 0.01, the uninsured were 593 % more likely to be transferred to another hospital. When a hospital dominates its market, it tends to expel uninsured patients to other hospitals.

**Conclusion:**

If patient transfers are medically unnecessary and primarily due to financial incentives, health administrators and policymakers should minimize such events. Since the uninsured who are admitted to a hospital that dominates its hospital market are likely to be much more vulnerable in their access to health care services, the state government of Florida needs to move toward increased health insurance coverage for eligible Floridians.

## Background

Among all Americans, 54.9 % have their health insurance coverage through employer-sponsored programs and 32.6 % are through government programs such as Medicare, Medicaid, SCHIP and CHAMPVA [1]. Although the coverage for non-elderly Americans increased between 2011 and 2012 [2], the number of the uninsured was still over 47 million in the U.S. Unlike other developed countries, there is no national health insurance program in the U.S. Instead, a majority of Americans have coverage through their employers. Even if they are offered coverage, however, a large number of employees cannot afford their share of the premiums because of the high cost [3].

More specifically, Florida has the second highest uninsured rate in the nation. In 2013, a total of 3.8 million people were uninsured and this represents about 25 % of the state’s population [4]. The Affordable Care Act (ACA) was passed in 2010 and it provided Florida a great opportunity to cover a substantially large proportion of the uninsured. In June 2012, however, the Federal Supreme Court ruled that each state is responsible for implementing Medicaid expansion to cover the low-income uninsured in the state. Republican Governor Scott and the Republican-led Legislature have relentlessly expressed their opposition to the ACA. Even as of today, they are not planning to implement Medicaid expansion. Although more than 440,000 Floridians signed up for health insurance under the ACA during the 6-month enrollment period, if the state had allowed Medicaid expansion, the number of uninsured would have been reduced at a much faster rate.

The consequences of being uninsured are well documented in the literature. For instance, the uninsured have less access to care, including preventive care, and chronic disease management [5–7]; they are at higher risk for preventable hospitalizations and have higher chances of missed diagnoses of serious conditions [8]; they have fewer follow-up visits, delayed subsequent care, and significantly higher mortality rates [9]; and they have less of a chance of receiving high-tech care [10]. For these reasons, the primary goal of the ACA is to lower the number of uninsured by providing affordable health insurance to low-income Americans.

Although it is a less spotlighted issue compared to the previous mentioned problems, an equally important topic is the likelihood of inter-hospital patient transfers between the insured and the uninsured. Various studies have found that insurance status influences inter-hospital patient transfer patterns [11–14].

Public hospitals and academic medical centers may admit more poorly insured transfer patients than do other institutions. Researchers have investigated the relationship between patient insurance status and inter-hospital patient transfers. Using the 2000 inpatient discharge data from California, Green et al. [14] revealed that a patient’s insurance status affected the likelihood of a hospital admitting a transfer patient. After dividing patients into good payer and poor payer patients based on the expected level of insurance reimbursement, they concluded that good payer patients were more likely to be transferred than were poor payer patients. Another study by Babu et al. [11], using the 2002 to 2006 American College of Surgeons National Trauma Databank, showed that uninsured patients are more likely to be transferred out of a Level II or III facility and are less likely to be accepted by a Level II or III facility for transfer compared with privately insured patients. By employing the 1991 data on all general acute care neo-pediatric hospitals in five counties in Pennsylvania, Durbin et al. [12] found that uninsured infants were twice as likely to be transferred compared to privately insured infants even after controlling for prematurity, severity of illness, and the level of neonatal intensive care units in the referring hospital. Recently, based on the comparison between transferred and nontransferred patients with a primary hand diagnosis at two trauma referral centers in Boston, Massachusetts, Eberlin et al. [13] revealed that the number of the uninsured was greater for transferred patients.

Presently, there are very few studies that have examined the relationship between hospital competition and inter-hospital patient transfers. Using data on 35 hospitals in an Italian region from 2003 to 2007, Mascia et al. [15] revealed that an increase in inter-hospital competition (i.e., measured by the overlap of hospitals using common resources) is associated with an increase in the number of patients transferred between dyads of hospitals. Another study that examined the relationship between hospital competition and inter-hospital transfers was done by Lomi and Pallotti [16]. By examining patient transfer relations among members of a community of hospital organizations located in Lazio, Italy, they found that hospitals are more likely to transfer patients when they are competing more intensely for patients across multiple geographical segments of their market.

Unfortunately, there has been no study that examines the relationship between hospital competition and inter-hospital patient transfers using U.S. data. Based on previous studies, a general consensus is that when a hospital is located in a more competitive market (i.e., having multiple competitors and/or a prominent competitor), it is more likely to admit less lucrative patients due to market pressures. When a hospital is located in a less competitive market (i.e., the hospital dominates its hospital market), however, it has more leverage to select out of its business those patients who are less profitable. It is a well-known fact that admitting the uninsured is financially risky because there is a higher chance that they will not pay the bill. Based on this perspective, it could be possible to infer that uninsured patients are more vulnerable to inter-hospital transfers. Another perspective would be that when a hospital dominates its market, especially if the hospital is nonprofit or public, it then admits more uninsured patients in order to show that it is doing its job.

In sum, the main objective of this study is to examine how hospital competition affects patient-sharing networks for different payer groups. By developing the payer-specific level of hospital competition, this research evaluates the effect of hospital competition on inward and outward patient sharing (or patient transfers) for different types of payers including the uninsured and also provides an understanding of the health equity issues surrounding the market structure of hospitals and the insurance status of patients in particular within Florida, USA.

## Methods

### Data

There are two data sources from the Florida Agency for Health Care Administration for this study: 1) the 2010 Hospital Inpatient Discharge Data, which includes all the information about inpatient discharges; and 2) the 2010 Hospital Financial Data Book, which contains general hospital characteristics. The unit of analysis is the general acute-care hospital in Florida.

### Construction of variables and statistical analyses

The dependent variables are the dyadic payer-specific patient-sharing relations between hospitals, which are measured by counting the number of transfers considering both inward patient sharing (i.e., the transfers from other hospitals to focal hospitals) and outward patient sharing (i.e., the transfers from focal hospitals to other hospitals). Instead of focusing on whether a specific patient is transferred from one hospital to another hospital at the patient level [11–14], we measure the numbers of patient transfers between hospitals (both inward and outward) at the hospital level [17–19]. More specifically, to measure such dyadic relations, we traced all the patient transfers with the same patient IDs between hospitals, which occurred within the same or the immediate following day, and counted the numbers of inward and outward transfers by considering their directions separately. These dependent variables—the numbers of outward and inward patient transfers by the principal payers—are count variables that show enormously zero-inflated distributions. Due to the zero-inflated distributions, we employed a standard count data model such as either a negative binomial regression model or a Poisson regression model based on each model’s over-dispersion parameter [20, 21]. The negative binomial regression assumes that the dependent count variable *y* follows a negative binomial distribution given the independent variables *x*:

$$ P\left(y=k\Big|x\right) = \frac{\varGamma \left(k+\frac{1}{\alpha}\right)}{\varGamma \left(k+1\right)\varGamma \left(\frac{1}{\alpha}\right)}\frac{\left(\alpha {\upmu}^k\right)}{{\left(1+\alpha \upmu \right)}^{k+1/\alpha }},\ k=0,\kern0.5em 1,\kern0.5em 2, \dots, $$

where α is an overdispersion parameter. The statistical significance of the estimated coefficient α determines the choice between the negative binomial model and the Poisson model: if α is significantly different from zero (0), the utilization of the negative binomial regression analysis is correct.

The primary independent variable is payer-specific hospital competition. To measure payer-specific hospital competition, we adopted a model used by Zwanziger and Melnick [22]. Instead of measuring hospital competition within an arbitrarily defined hospital market (i.e., geopolitical boundaries such as cities and counties), we employed the Hirschman-Herfindahl Index (HHI) as a measure of hospital competition. The payer-specific HHIs we used were based on the patient-origins data by patient zip code in each of the payer groups to determine the extent of each hospital’s market [22] for specific payer groups. The payer-specific HHIs for a hospital market is the sum of the squared market shares for all the hospitals competing in the market for each payer group. The HHIs range from 0 to 1, with lower levels of HHI indicating greater competition.

For control variables, we used hospital ownership (i.e., for-profit, non-profit, and public), location (i.e., urban vs. rural), teaching status (i.e., teaching vs. non-teaching), hospital case mix index (CMI), total number of discharges, average length of stay, occupancy rate, and the difference between revenue and cost per adjusted admission.

With the variables constructed as shown above, we developed two models to examine the effects of competition among hospitals on patient sharing: one for outward transferred patients from focal hospitals and the other for inward transferred patients to focal hospitals. The Incidence Rate Ratio (IRR), which is used to report the Poisson or negative binomial regression results, represents a change in the dependent variable in terms of a percentage increase or decrease, with the percentage determined by the amount that the IRR is either above or below one (1) [23]. For example, an IRR reporting (1.32) would be written as a 32 % increase ((a value 0.32 more than 1) × 100) with every one unit increase in an independent variable. An IRR reporting (0.68) would be written as a 32 % decrease ((a value 0.32 less than 1) × 100) with every one unit increase in an independent variable.

## Results

### Descriptive statistics and visualizations of inter-hospital patient transfers

As Table 1 indicates, among the 172 hospitals located in Florida, 155 hospitals had Medicare, Medicaid, Commercial health insurance, and uninsured patients; 153 hospitals had Medicare managed care patients; and 146 hospitals had Medicaid managed care patients. Each hospital, on average, transferred 6.82 patients in total: 1.61 Medicare patients; .35 Medicare Managed Care patients; 1.58 Medicaid patients; .23 Medicaid Managed Care patients; 1.05 commercial health insurance patients; and .20 uninsured patients. Hospitals in Florida confront the low level of competition with the mean HHIs ranging from .42 for commercial health insurance to .58 for Medicaid Managed Care. On average, almost half of the hospitals are for-profits; 5 % of the hospitals are involved in teaching.

**Table 1:** Descriptive statistics

Variables	Obs.	Mean	Std. Dev.	Min	Max
Total outward patients	155	6.82	17.74	0	150
Total inward patients	155	6.82	16.16	0	150
Medicare outward patients	155	1.61	4.95	0	44
Medicare inward patients	155	1.61	3.73	0	36
Medicare managed care outward patients	153	.35	1.11	0	10
Medicare managed care inward patients	153	.35	.85	0	5
Medicaid outward patients	155	1.58	5.19	0	39
Medicaid inward patients	155	1.58	5.40	0	52
Medicaid managed care outward patients	146	.23	.79	0	5
Medicaid managed care inward patients	146	.23	.72	0	4
Commercial health insurance outward patients	155	1.05	3.01	0	28
Commercial health insurance inward patients	155	1.05	2.76	0	23
Uninsured outward patients	155	.20	.99	0	10
Uninsured inward patients	155	.20	.69	0	6
Total HHI	155	.42	.10	.20	.84
Medicare HHI	155	.47	.11	.21	.76
Medicare managed care HHI	153	.53	.12	.26	1
Medicaid HHI	155	.48	.10	.26	.85
Medicaid managed care HHI	146	.58	.14	.32	1
Commercial Health Insurance HHI	155	.42	.09	.25	.80
Uninsured HHI	155	.55	.11	.33	.91
For profit	155	.50	^a^	0	1
Non-profit	155	.39	^a^	0	1
Public	155	.09	^a^	0	1
Teaching	155	.05	^a^	0	1
Rural	155	.12	^a^	0	1
CMI	155	1.32	.21	.72	2.14
Total # of discharges	155	14986.8	15802.9	223	127089
Average length of stay	155	3.40	.52	2.24	6.20
Occupancy rate	155	54.33	15.92	5.16	87.66
(Revenue per adjusted admission)-(Cost per adjusted admission)	155	5958.6	1659.4	3413.9	12957.6

*Note*: ^a^designates binary variable (0, 1)

**Fig. 1:**
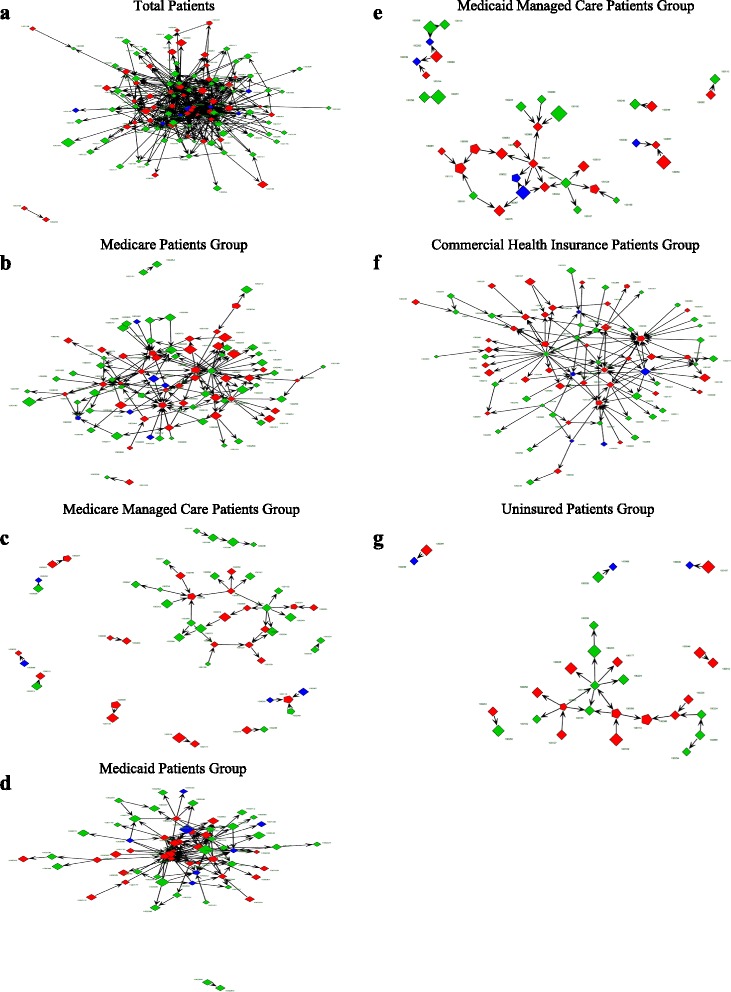
Payer-Specific patients transfer networks

We also provide a visualization of the payer-specific patient-sharing networks to improve our understanding of the characteristics inherent within inter-hospital relationships by using several attributive variables from each hospital (see Fig. 1). In each patient-sharing network, the size of the nodes is proportional to each hospital’s total and payer-specific HHIs. The arrowheads around the nodes indicate the direction of the patient transfers between two hospitals. The colors of the nodes are based on each hospital’s ownership: green for profits; red for non-profits; and blue for public hospitals. The shapes of the nodes are based on the hospital’s teaching status: squares for non-teaching and pentagons for teaching hospitals.

### Effects of hospital competition on patient transfers

As mentioned previously, we evaluated the effects of competition among hospitals on patient sharing with two models: one for outward transferred patients from focal hospitals (see Table 2) and the other for inward transferred patients to focal hospitals (see Table 3).

**Table 2:** The effects of competition among hospitals on the outward patients sharing

	Principal payer						
	Total	Medicare	Medicare managed care	Medicaid	Medicaid managed care	Commercial health insurance	Uninsured
	IRR (SE)^a^	IRR (SE)^a^	IRR (SE)^a^	IRR (SE)^a^	IRR (SE)^a^	IRR (SE)^a^	IRR (SE)^b^
Total HHI	.17 (.31)						
Medicare HHI		.02^**^ (.03)					
Medicare Managed Care HHI			.02 (.06)				
Medicaid HHI				1.17 (2.80)			
Medicaid Managed Care HHI					.17 (.47)		
Commercial Health Insurance HHI						3.04 (5.89)	
Uninsured HHI							594.82^**^ (1570.31)
For Profit (0 = Public)	2.94^*^ (1.64)	3.98^*^ (3.08)	1.14 (.81)	1.97 (1.75)	.49 (.46)	2.36 (1.73)	2.63 (2.68)
Non-profit (0 = Public)	1.82 (.99)	3.01 (2.26)	.71 (.48)	1.34 (1.10)	1.13 (.99)	2.51 (1.68)	.35 (.34)
Teaching (0 = Non-teaching)	.27^*^ (.20)	1.29 (1.14)	4.87^**^ (3.43)	.24 (.24)	.23 (.29)	.76 (.55)	13.69^***^ (7.78)
Rural (0 = Urban)	1.67 (.98)	6.89^***^ (4.74)	1.23 (1.48)	1.12 (1.45)	.00 (.00)	.00 (.00)	.00 (.00)
CMI	215.18^***^ (277.66)	534.67^***^ (811.82)	45.72^**^ (74.87)	96.77^**^ (197.13)	35.79 (77.84)	40.34^***^ (55.12)	303.1^**^ (680.84)
ln(Total # of Discharges)	1.0001^***^ (.00)	1.0001^***^ (.00)	1.00004^***^ (.00)	1.0001^***^ (.00)	1.00004^**^ (.00)	1.00005^***^ (.00)	1.0001^***^ (.00)
Average Length of Stay	2.84^**^ (1.30)	2.63^*^ (1.41)	1.48 (.84)	1.37 (.89)	5.33^*^ (4.58)	2.52^*^ (1.23)	6.47^***^ (4.44)
Occupancy Rate	1.01 (.01)	1.02^*^ (.01)	1.01 (.01)	.99 (.01)	1.01 (.02)	1.00 (.01)	1.03 (.02)
(Revenue per Adjusted Admission) – (Cost per Adjusted Admission)	.99 (.00)	.99^***^ (.00)	.99^**^ (.00)	1.00 (.00)	.99 (.00)	.99 (.00)	.99^**^ (.00)
*Overdispersion Parameter*	2.14^***^ (.34)	1.90^***^ (.48)	.65^**^ (.46)	3.49^***^ (.90)	1.85^***^ (1.07)	1.44^***^ (.41)	
*Model χ* ^*2*^	85.95	69.41	45.63	54.25	29.43	67.88	121.66
*df*	10^c^	10^c^	10^c^	10^c^	10^c^	10^c^	10^c^
*Pseudo R* ^*2*^	.11	.15	.20	.14	.18	.17	.57
*N*	155	155	153	155	146	155	155

*Notes*: *p < .1, **p < .05, ***p < .01

^a^Negative binomial regression model

^b^Poisson regression model

^c^Compared to model with intercept only

**Table 3:** The effects of competition among hospitals on the inward patients sharing

	Principal payer						
	Total	Medicare	Medicare managed care	Medicaid	Medicaid managed care	Commercial health insurance	Uninsured
	IRR (SE)^a^	IRR (SE)^a^	IRR (SE)^a^	IRR (SE)^a^	IRR (SE)^a^	IRR (SE)^a^	IRR (SE)^a^
Total HHI	1.40 (2.09)						
Medicare HHI		4.60 (6.76)					
Medicare Managed Care HHI			.02 (.06)				
Medicaid HHI				2.12 (4.52)			
Medicaid Managed Care HHI					.02 (.08)		
Commercial Health Insurance HHI						1.90 (3.99)	
Uninsured HHI							.43 (1.27)
For Profit (0 = Public)	1.10 (.50)	2.25 (1.34)	1.14 (.81)	.53 (.36)	.16^*^ (.15)	1.28 (.87)	.89 (.73)
Non-profit (0 = Public)	1.21 (.55)	2.78^*^ (1.58)	.71 (.48)	.53 (.37)	.48 (.39)	1.63 (1.02)	.40 (.36)
Teaching (0 = Non-teaching)	.55 (.37)	2.13 (1.69)	4.87^**^ (3.43)	.38 (.37)	1.60 (2.19)	.68 (.56)	1.40 (1.69)
Rural (0 = Urban)	.92 (.45)	1.29 (.79)	1.23 (1.48)	.80 (.77)	.00 (.00)	.26 (.30)	1.95 (2.18)
CMI	63.94^***^ (73.79)	70.92^***^ (88.77)	45.72^**^ (74.87)	39.64^**^ (69.25)	13.24 (32.77)	26.04^**^ (37.10)	25.99 (59.35)
ln(Total # of Discharges)	1.0001^***^ (.00)	1.00003^**^ (.00)	1.00004^***^ (.00)	1.0001^***^ (.00)	1.0001^*^ (.00)	1.0001^***^ (.00)	1.00 (.00)
Average Length of Stay	3.02^***^ (1.28)	3.28^**^ (1.63)	1.48 (.84)	1.62 (.88)	3.22 (2.87)	1.82 (.88)	2.39 (1.83)
Occupancy Rate	1.01 (.01)	1.01 (.01)	1.01 (.01)	1.01 (.01)	1.01 (.02)	1.00 (.01)	1.03 (.01)
(Revenue per Adjusted Admission) – (Cost per Adjusted Admission)	.99^***^ (.00)	.99^***^ (.00)	.99^**^ (.00)	.99 (.00)	.99 (.00)	.99 (.00)	.99 (.00)
*Overdispersion Parameter*	1.68^***^ (.26)	1.56^***^ (.25)	.65^**^ (.46)	2.38^***^ (.56)	2.48^***^ (1.28)	1.70^***^ (.45)	2.92^***^ (1.52)
*Model χ* ^*2*^	84.75	57.08	45.63	62.37	26.95	55.30	13.55
*df*	10^b^	10^b^	10^b^	10^b^	10^b^	10^b^	10^b^
*Pseudo R* ^*2*^	.10	.11	.20	.14	.16	.14	.08
*N*	155	155	153	155	146	155	155

*Notes*: *p < .1, **p < .05, ***p < .01

^a^Negative binomial regression model

^b^Compared to model with intercept only

Controlling for the variables regarding hospital characteristics, hospitals were 59,382 % (IRR = 594.82, *p* < .05) more likely to transfer uninsured patients to other hospitals with every one unit increase in uninsured HHI. As mentioned earlier, HHIs range from 0 to 1. More realistically, therefore, as the uninsured HHI increases by 0.01, the uninsured were 593 % more likely to be transferred to another hospital. For the uninsured, when a hospital is located in a less competitive market, outward patient sharing increases. That is, when a hospital dominates the market, it tends to expel uninsured patients to other hospitals.

Holding all other hospital-related attributes constant, hospitals were 98 % (IRR = .02, *p* < .05) less likely to transfer Medicare patients to other hospitals with every one unit increase in Medicare HHI. As the Medicare HHI increases by 0.01, Medicare patients are .98 % less likely to be transferred to another hospital. For Medicare patients, when a hospital is located in a less competitive market, outward patient sharing decreases. That is, when a hospital dominates the market, it tends to retain more Medicare patients.

## Discussion and conclusions

In 2010, there were about 1.46 million inpatient discharges in 155 general acute care hospitals in Florida. Among those discharges, there were only 1059 patient transfers between hospitals. If we had employed a looser definition of patient transfers, the number would have been higher than 1059. Even if there had been a smaller number of patient transfers, this study is a first attempt to understand how hospital competition affects patient transfer activities using a dyadic relationship between hospitals. As mentioned earlier, the Florida hospital market is less competitive (i.e., HHIs range from .42 to .58) even if we look at the different payers. In a such hospital market, it is unclear how hospitals behave in relation to patient sharing. This study reveals that when a hospital is located in a less competitive market, or when a hospital is dominating its market, it tends to transfer uninsured patients to another hospital. Under the same conditions, on the other hand, a hospital is less likely to transfer Medicare patients from its facility to another hospital. That is, beyond the relationship between insurance status and inter-hospital transfers examined in the previous studies [11–14], we could show differentiated relationships between the market structure of the hospital and patient transfers based on their insurance status. As our findings indicate, the uninsured who are admitted to a hospital that dominates its hospital market are likely to be much more vulnerable in their access to health care services, requiring the state government of Florida to move toward increased health insurance coverage for eligible Floridians.

It is unclear the exact reason for patient transfers especially for the different patterns of patient transfers among different payers. Thus, it is important to discern whether or not patient transfers are medically necessary. If such transfers are medically necessary, they should be promoted and recommended. If they are medically unnecessary and primarily due to financial incentives relating to the market structure in which the hospitals provide health services and the insurance status of the patients, health administrators and policymakers should minimize such events. Also, since there is little knowledge about the underlying professional, financial, or personal incentives for hospital admission staff and administrators regarding inter-hospital patient transfer decisions, this suggests a direction for future research.

With the expansion of managed care, hospitals have provided significant discounts to those insured by Medicare, Medicaid, and commercial insurers. However, they have not discounted their charges and in fact have even raised them for the uninsured because there have been no negotiations between the uninsured and the hospitals. In order to protect the uninsured from such high prices, in 2006 California adopted a fair pricing law that prohibits hospitals from charging the uninsured the full amounts or significantly higher fees. Five years after the inception of this fair pricing law, most Californian hospitals have been equipped with financial assistance programs for the uninsured [24] and the uninsured have been asked to pay a lower amount of the hospital charges.

Although Florida has not had such a law that protects the uninsured, the recent health care reform under the ACA provided an opportunity to cover those who are currently uninsured. Yet, the current Scott Administration in Florida has opposed the Medicaid expansion program that would have insured the low-income uninsured. Even though Florida’s Low Income Pool (LIP) health-funding program has been recently extended for two additional years [25], the funds help hospitals cover the costs of treating uninsured and underinsured patients after the fact, rather than increasing eligible Floridians’ health insurance coverage as the Medicaid expansion program would have done. If the uninsured had been covered by the Medicaid expansion program, they would not have been transferred to other hospitals mainly due to financial reasons as our findings indicate. It is too early to predict what will happen in Florida in terms of covering the uninsured. If they remain uninsured, however, they are more likely to be transferred to other hospitals, especially when they are admitted to a hospital that dominates its hospital market.

As managed care promotes a lower cost of providing health care services while maintaining a high quality of care, similar to other states, Florida has attempted to increase the enrollment of its Medicaid managed care programs. As a result, Medicaid enrollment grew 3.4 times faster than its population growth during the period between 1990 and 2010. Along with the increase in Medicaid enrollment, Florida’s Medicaid expenditures increased at an annual rate of 10.1 % during the same time period. Based on other states’ experiences (such as New York), as Medicaid enrollment increases, the level of competition that hospitals encounter increases. In such a situation, the difference between allowable charges and costs would become smaller and smaller. If the uninsured remain uninsured, the financial pressures the uninsured would experience would be greater because hospitals would charge them higher prices.

Certainly, there are necessary patient transfers based on medical reasons. If it is necessary to transfer a patient to another facility based on medical reasons, it should be promoted. Due to the data limitations, however, we were unable to examine patient sharing from a perspective of “from which hospital to which hospitals based on medical reasons.” Furthermore, we expect that the construction of a longitudinal network dataset of inter-hospital patient transfers in future research will allow us to determine what kinds of dyadic characteristics between hospitals motivate or incentivize hospitals to transfer patients to each other.
